# Identification of microRNA signature in the progression of gestational trophoblastic disease

**DOI:** 10.1038/s41419-017-0108-2

**Published:** 2018-01-24

**Authors:** Jiu-Ru Zhao, Wei-Wei Cheng, Ya-Xin Wang, Mei Cai, Wei-Bin Wu, Hui-Juan Zhang

**Affiliations:** 1 Departments of Pathology and Bio-Bank, International Peace Maternity and Child Health Hospital, Shanghai Jiao Tong University School of Medicine, Shanghai, 200030 China; 2 Institute of Embryo-Fetal Original Adult Disease, Shanghai Jiao Tong University School of Medicine, Shanghai, 200030 China; 3 Department of Obstetrics, International Peace Maternity and Child Health Hospital, Shanghai Jiao Tong University School of Medicine, Shanghai, 200030 China; 4 Department of Ultrasound in Medicine, Shanghai Jiao Tong University Sixth People’s Hospital, Shanghai, 200233 China

## Abstract

Gestational trophoblastic disease (GTD) encompasses a range of trophoblast-derived disorders. The most common type of GTD is hydatidiform mole (HM). Some of HMs can further develop into malignant gestational trophoblastic neoplasia (GTN). Aberrant expression of microRNA (miRNA) is widely reported to be involved in the initiation and progression of cancers. MiRNA expression profile also has been proved to be the useful signature for diagnosis, staging, prognosis, and response to chemotherapy. Till now, the profile of miRNA in the progression of GTD has not been determined. In this study, a total of 34 GTN and 60 complete HMs (CHM) trophoblastic tissues were collected. By miRNA array screening and qRT-PCR validating, six miRNAs, including miR-370-3p, -371a-5p, -518a-3p, -519d-3p, -520a-3p, and -934, were identified to be differentially expressed in GTN vs. CHM. Functional analyses further proved that miR-371a-5p and miR-518a-3p promoted proliferation, migration, and invasion of choriocarcinoma cells. Moreover, we demonstrated that miR-371a-5p was negatively related to protein levels of its predictive target genes *BCCIP, SOX2*,* and BNIP3L*, while miR-518a-3p was negatively related to *MST1* and *EFNA4*. For the first time, we proved that miR-371a-5p and miR-518a-3p directly targeted to 3′-UTR regions of *BCCIP* and *MST1*, respectively. Additionally, we found that miR-371a-5p and miR-518a-3p regulated diverse pathways related to tumorigenesis and metastasis in choriocarcinoma cells. The results presented here may offer new clues to the progression of GTD and may provide diagnostic biomarkers for GTN.

## Introduction

Gestational trophoblastic disease (GTD) is a group of diseases originating from trophoblastic cells with abnormal proliferation and metastasis. GTD heterogeneously comprises gestational trophoblastic neoplasia (GTN), hydatidiform mole (HM), benign non-neoplastic lesions, and villous lesion^1,2^. HM, the most common member of GTD, includes two entities: partial hydatidiform mole (PHM) and complete hydatidiform mole (CHM). PHM is characterized by partial edema and focal trophoblastic proliferation in chorionic villi, while CHM presents exaggerated proliferation of abnormal trophoblast in nearly every villus. PHM is genetically almost triploid with one maternal chromosome set and two paternal chromosome sets while CHM is generally androgenetic diploid^3^. Incidence of GTD could reach up to 2.0 per 1000 pregnancies^4^. Generally, most HMs would regress after uterine evacuation, whereas about 15%–20% of CHM cases and 0.5% of PHM cases could progress into GTN^5,6^. Clinically, persistently raised or rising serum human chorionic gonadotropin (hCG) level after evacuation of HM is an indication of GTN. Although GTN patients showed more than 90% cure rate with a good response to chemotherapy, around 4% cases would succumb to chemotherapy^7^. Currently, the exact molecular mechanisms of etiopathogenesis and progression of GTD still remain largely unknown.

MicroRNAs (miRNAs) are 19–24 nucleotides small non-coding RNAs that participate in all fundamental cellular processes, involving in the regulation of translation and degradation of nearly 50% of the human mRNAs^8^. Aberrant expression of miRNAs has been widely detected in human cancers^9^. For example, Iorio et al. identified 29 dysregulated miRNAs in breast cancer^10^, Lee et al. identified 112 aberrantly expressed miRNAs in pancreatic cancer^11^, and Yanaihara et al. discovered 43 differentially expressed miRNAs in lung cancer^12^. Besides, increasing evidences indicated that miRNAs dysregulation could be an early event that occurred in precancerous stage^13^. Many miRNAs have been reported to be signatures of cancer prognosis, such as miR-15a and miR-16-1 in chronic lymphocytic leukemia, miR-143 and miR-145 in colorectal cancer, let-7 in lung cancer, miR-155 in diffuse large B-cell lymphoma, miR-221 and miR-222 in papillary thyroid carcinoma, and miR-375 in laryngocarcinoma^14^. Moreover, miRNAs have been proved to be a more accurate marker for the classification of cancer subtype than mRNAs, and several miRNAs are common signatures for different cancers. For example, miR-21 was reported to be overexpressed in six types of cancers, and miR-17-5p and miR-191 were overexpressed in five^15^.

So far, genome-wide analysis has identified more than 2500 human miRNAs, including over 700 miRNAs in human placenta. Several miRNAs are trophoblast-specific, such as the most intriguing family—chromosome 19 miRNA cluster (C19MC), which contains 46 intronic miRNA genes that are scattered over 100 kb of genomic DNA and produces 58 mature miRNAs^16^. Although numerous progresses in placenta-associated miRNAs have been made, studies about miRNAs in the pathogenesis and progression of GTD are still rudimentary. Previously, Na et al. reported that miR-517a, -517b, -518b, and -519a were dysregulated in CHM^17^. Hasegawa et al. found high levels of miR-520b, -520f, and -520c-3p significantly decreased in plasma of CHM patients after evacuation^18^. Miura et al. further demonstrated that these three miRNAs showed similar variation tendency to serum hCG concentration in GTN patients^19^. Chao et al. discovered that miR-199b was downregulated in human choriocarcinoma cells compared with normal trophoblasts^20^. Previously, we also discovered that miR-21 was overexpressed in GTN^21^. However, most of these studies were based on very small sample sizes, making these results less conclusive. Systemic investigation about the expression profile and roles of miRNAs in GTD is still needed.

In the present study, we aimed to explore the differentially expressed miRNAs in GTN vs. CHM. Further, we sought to determine the roles of dysregulated miRNAs in proliferation and metastasis of trophoblastic cells, and to elucidate the molecular regulation networks. Our results would be helpful for the investigation of early molecular biomarkers and therapeutic targets for GTN.

## Results

### Altered miRNA expression profile in GTN vs. CHM

**Fig. 1:**
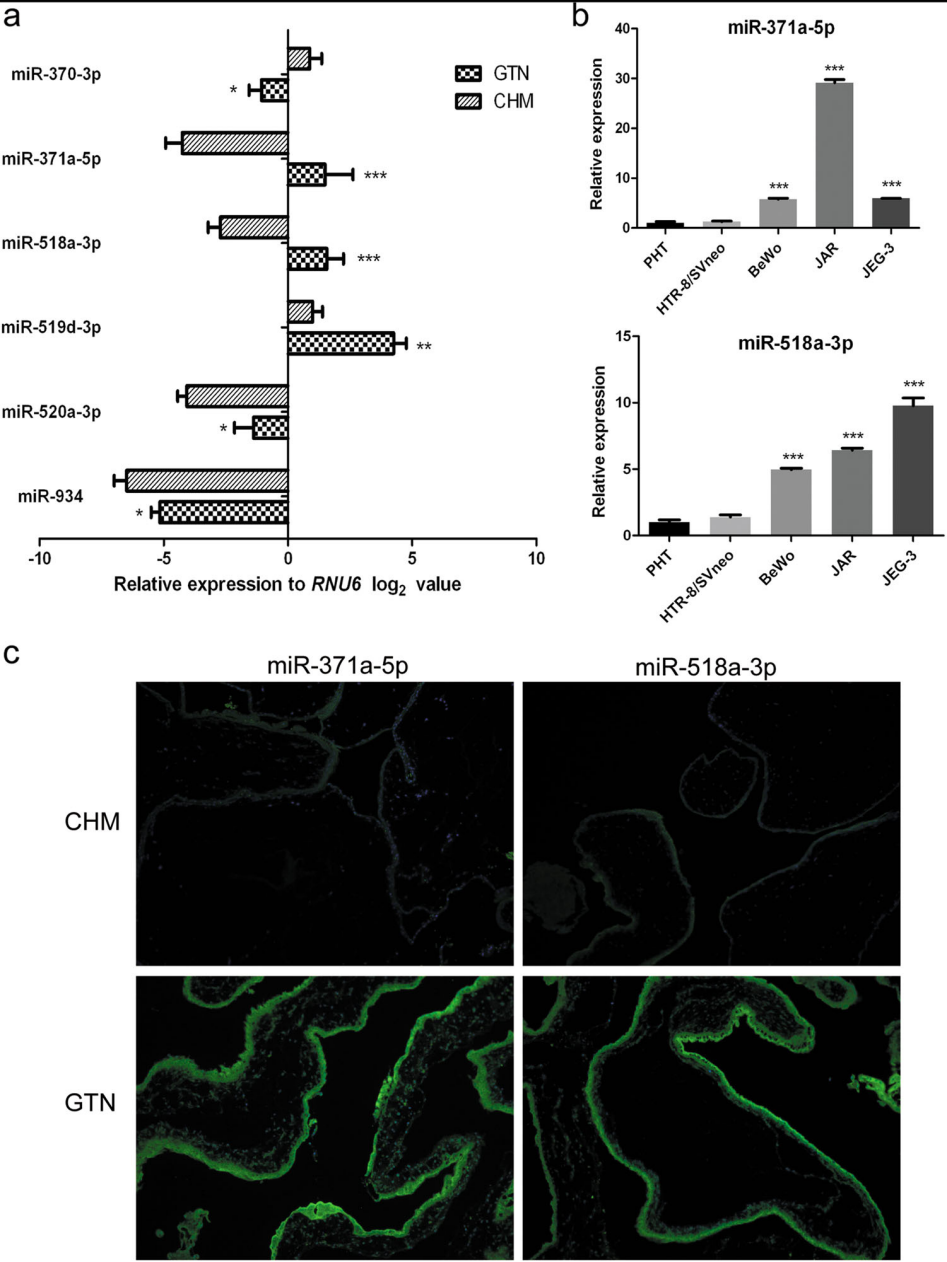
MiR-371a-5p and miR-518a-3p were upregulated in GTN tissues and choriocarcinoma cells **a** Validation of the microarray profiling data. Expression levels of the most differentially expressed 16 miRNAs were examined by qRT-PCR in 34 cases of GTN and 60 cases of CHM trophoblastic tissues and then normalized to the housekeeping gene *RNU6* with the 2^−ΔΔCt^ method. Differences were evaluated by using Mann–Whitney *U*-test. Fold changes were 0.26 for miR-370-3p, 217.56 for miR-371a-5p, 19.5 for miR-518a-3p, 9.63 for miR-519d-3p, 6.41 for miR-520a-3p, and 2.52 for miR-934. **b** miR-371a-5p and miR-518a-3p expression levels quantified by qRT-PCR in villous primary human trophoblasts (PHT), immortalized normal trophoblastic cell line HTR-8/SVneo, and choriocarcinoma cell lines BeWo, JAR, and JEG-3. Relative expressions in choriocarcinoma cells were normalized against corresponding miRNA levels in PHT cells. * *P* < 0.05, ** *P* < 0.01, *** *P* < 0.001. **c** Representative micrographs of miR-371a-5p and miR-518a-3p detected with fluorescence in situ hybridization. Positive signals for miR-371a-5p or miR-518a-3p are visualized in green, while blue depicts DAPI nuclear stain. Magnification 100×

A small-scale miRNA microarray was performed to evaluate miRNA expression profiles in GTN, CHM, and normal first-trimester placentas. The ANOVA and *t*-tests were used to identify miRNAs whose expression were significantly different among these three different categories of trophoblastic tissues based on normalized data^10^. Totally, 119 upregulated and 134 downregulated miRNAs were identified (*P* < 0.05) in GTN tissues compared with CHM. Meanwhile, cluster analysis generated a tree with clear distinction between GTN and normal first-trimester placentas (Supplementary Figure S1a). Scatter plot analysis further indicated that there were several differentially expressed miRNAs between GTN and CHM (Supplementary Figure S1b). Sixteen miRNAs were found with over fivefold changes between GTN and CHM, including miR-10b-5p, -21, -98-5p, -99a-5p, -125b-2-3p, -196b-5p, -196b-3p, -370-3p, -371a-5p, -372-3p, -518a-3p, -518b, -519d-3p, -520a-3p, -584-5p, and -934. For validation, quantitative real-time polymerase chain reaction (qRT-PCR) test for these 16 miRNAs was carried out in fresh tissues (13 cases of GTN, 25 cases of CHM) and in formalin-fixed, paraffin-embedded (FFPE) tissues (21 cases of GTN, 35 cases of CHM). Notably, six miRNAs, including miR-370-3p, -371a-5p, -518a-3p, -519d-3p, -520a-3p, and -934, were identified to be differentially expressed in GTN vs. CHM (Fig. 1a). Among which, all, except miR-370-3p, were upregulated in GTN. Besides, relative expression levels of miR-371a-5p and miR-518a-3p, the two most differentially expressed miRNAs, were also detected in choriocarcinoma cells. Similar to the expression pattern in GTN, levels of both miRNAs were at least fivefolds higher in choriocarcinoma cells than in normal primary human trophoblastic cells (PHT) and HTR-8/SVneo cells (Fig. 1b, all *P* < 0.001). To address the cellular origin of miR-371a-5p and miR-518a-3p in trophoblastic tissues, in situ hybridization (ISH) was performed in the 56 FFPE tissues. As indicated in Fig. 1c, positive signals were detected predominantly in trophoblastic layers for both miRNAs, and were much stronger in GTN than in CHM. NBT/BCIP staining also revealed that the staining scores of both miRNAs were significantly higher in GTN than in CHM (Supplementary Figure S2, Tables S1 and S2). Considered together, our data indicated that miR-371a-5p and miR-518a-3p were upregulated in villous trophoblastic cells of GTN.

### MiR-371a-5p and miR-518a-3p promoted proliferation of choriocarcinoma cells

**Fig. 2:**
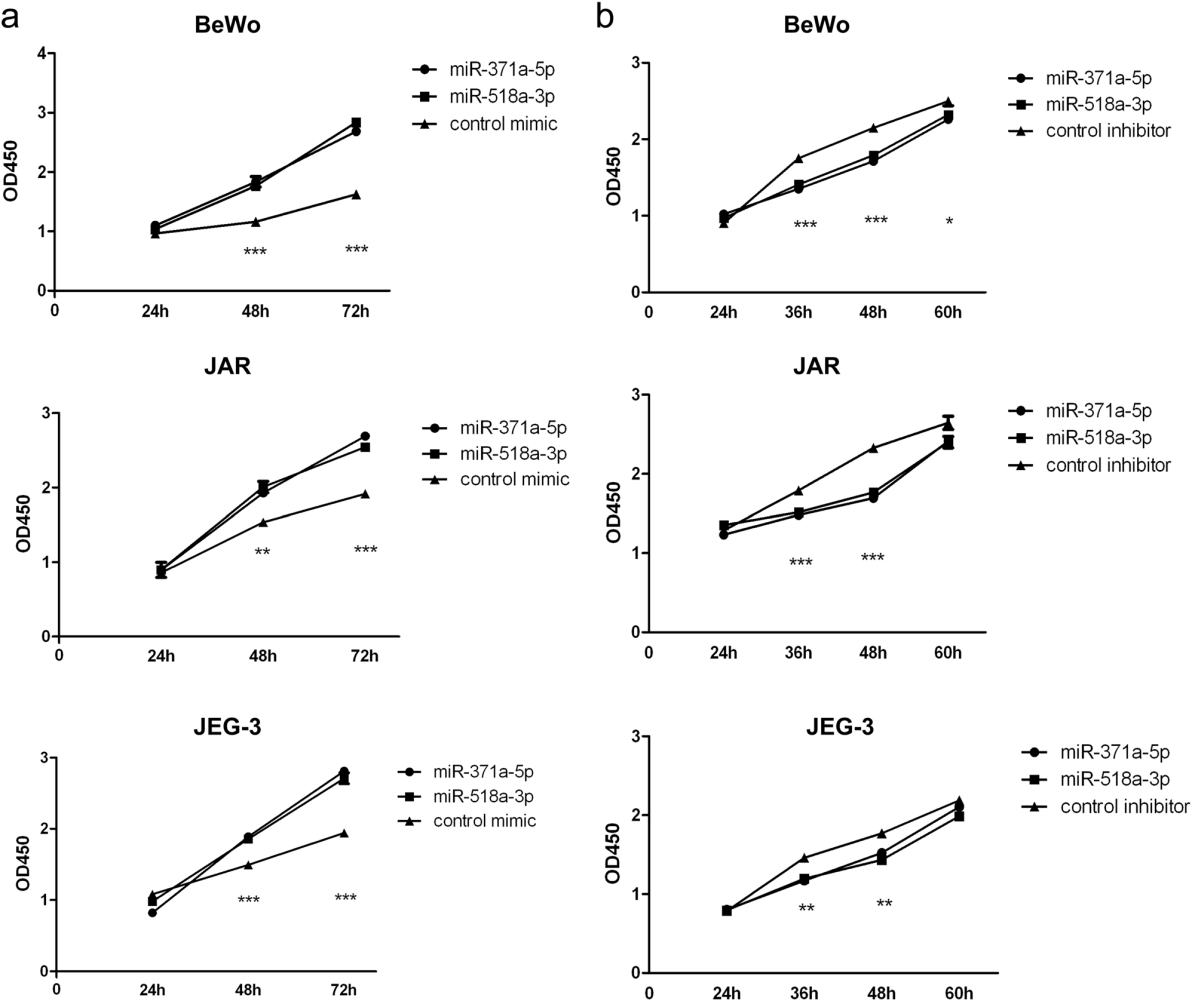
Overexpressions of miR-371a-5p and miR-518a-3p promoted proliferation of choriocarcinoma cells BeWo, JAR, and JEG-3 cells in 96-well plates were transfected with 30 nM miR-371a-5p or miR-518a-3p mimics (**a**, left) or 40 nM inhibitors (**b**, right). Cell viabilities were measured by CCK-8 assay at indicated time points post transfection. *N* = 12, *P < 0.05, **P < 0.01, ***P < 0.001

To investigate the functions of miR-371a-5p and miR-518a-3p in choriocarcinoma cells, firstly, BeWo, JAR, and JEG-3 cells were transiently transfected with corresponding miRNA mimics, inhibitors, or controls. qRT-PCR results indicated that overexpressions of miR-371a-5p and miR-518a-3p induced at least 50-folds and 150-folds increases in all three cell lines, respectively (Supplementary Figure S3a), while silencing of both miRNAs reduced their expression by more than 60% in all three cell lines (Supplementary Figure S3b). Then, cell viabilities were measured by CCK-8 assay at different time points post transfection (Fig. 2). Results indicated that overexpression of miR-371a-5p or miR-518a-3p significantly promoted proliferation of BeWo, JAR, and JEG-3 cells, whereas downregulation of miR-371a-5p or miR-518a-3p significantly inhibited proliferation of the three choriocarcinoma cells.

### Overexpression of miR-371a-5p or miR-518a-3p reduced the percentage of choriocarcinoma cells at the S phase of cell cycle

**Fig. 3:**
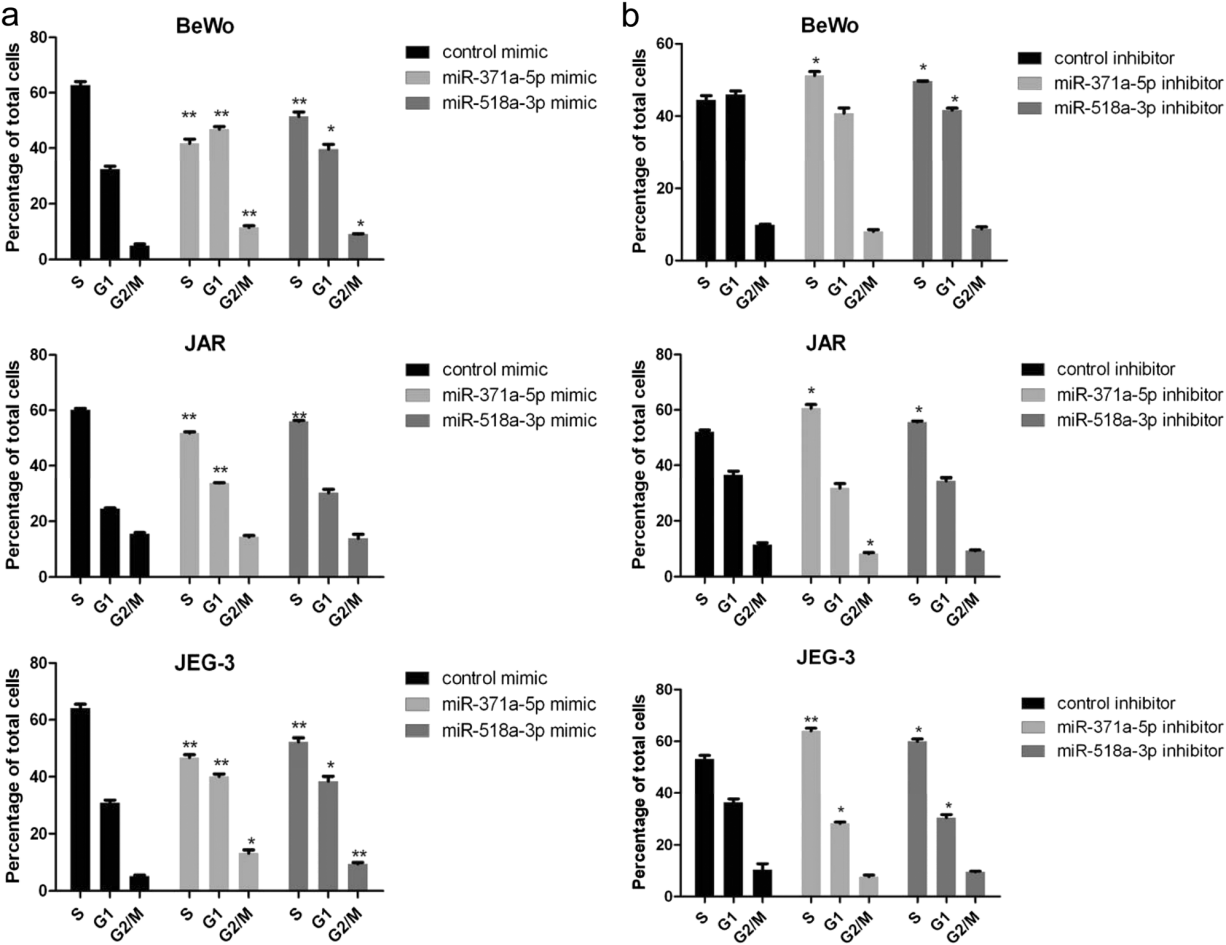
Downregulations of miR-371a-5p and miR-518a-3p arrested choriocarcinoma cells at the S phase of cell cycle BeWo, JAR, and JEG-3 cells in 24-well plates were transfected with 100 nM miR-371a-5p or miR-518a-3p mimics (**a**, left) or 150 nM inhibitors (**b**, right). Forty-eight hours post transfection, cells were stained with propidium iodide and quantified by flow cytometric analysis. *N* = 6, *P < 0.05, **P < 0.01

Forty-eight hours post transfection with mimics or inhibitors of miR-371a-5p and miR-518a-3p, the percentages of choriocarcinoma cells at each cell cycle phase were analyzed. Flow cytometric analysis (FCA) indicated that cells at the S phase were significantly reduced in all three choriocarcinoma cells with overexpressions of miR-371a-5p (0.66-fold in BeWo, 0.85-fold in JAR, 0.73-fold in JEG-3) or miR-518a-3p (0.82-fold in BeWo, 0.92-fold in JAR, 0.81-fold in JEG-3) (all *P* < 0.01). An increase in the percentages of cells at the G1 and G2/M phases was also observed in BeWo and JEG-3 cells post transfection with miR-371a-5p or miR-518a-3p mimics (Fig. 3a). In contrast, knockdown of both miRNAs arrested cell cycle at the S phase in all three cells (about 1.20-fold increase for miR-371a-5p and 1.1-fold increase for miR-518a-3p, both *P* < 0.05), thus hindered the entry to cell replication cycle (Fig. 3b).

### MiR-371a-5p and miR-518a-3p promoted migration and invasion of choriocarcinoma cells

**Fig. 4:**
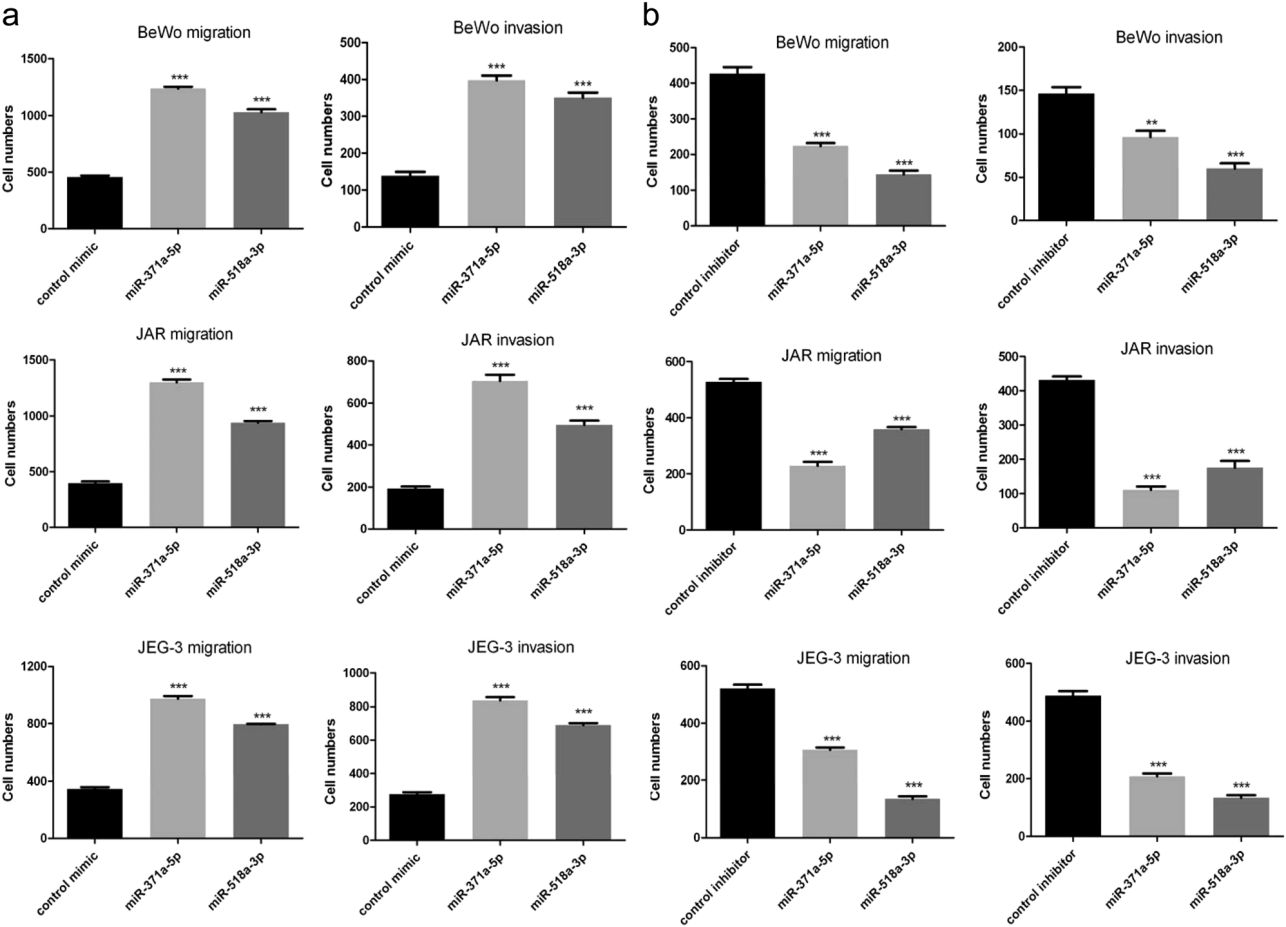
miR-371a-5p and miR-518a-3p promoted migration and invasion of choriocarcinoma cells BeWo, JAR, and JEG-3 cells were transfected with miR-371a-5p or miR-518a-3p mimics (**a**, left) or inhibitors (**b**, right). Twenty-four hours post transfection, cells were pretreated with mitomycin C and then harvested and seeded into transwell inserts with (for invasion) or without (for migration) Matrigel. After incubation, cells were fixed and stained, and then visualized under a microscope at 100× magnification. Five random selected fields were counted, and the average number was calculated for statistical analysis. *N* = 3, **P < 0.01, ***P < 0.001

Transwell assay was adopted to evaluate the effect of miR-371a-5p and miR-518a-3p on migration and invasion of choriocarcinoma cells. To eliminate the potential confounding effect of cell proliferation, mitomycin C was used^22^. Cell-number counting and CCK-8 assay revealed that 2 h pretreatment with 8 μg/ml mitomycin C for JEG-3 cells or 4 μg/ml mitomycin C for BeWo and JAR cells could block their proliferation within 36 h (data not shown). Thus, cells were pretreated with mitomycin C for 2 h before transwell assay. Marked increases in the number of migrated or invaded cells were observed in the miR-371a-5p or miR-518a-3p overexpressed groups (Fig. 4a and Supplementary Figure S4, all >2-folds, *P* < 0.001). Conversely, significant decreases in the number of migrated or invaded cells were detected in the miR-371a-5p or miR-518a-3p knockdown groups (Fig. 4b and Supplementary Figure S5).

### Validation of miR-371a-5p and miR-518a-3p potential targets

**Fig. 5:**
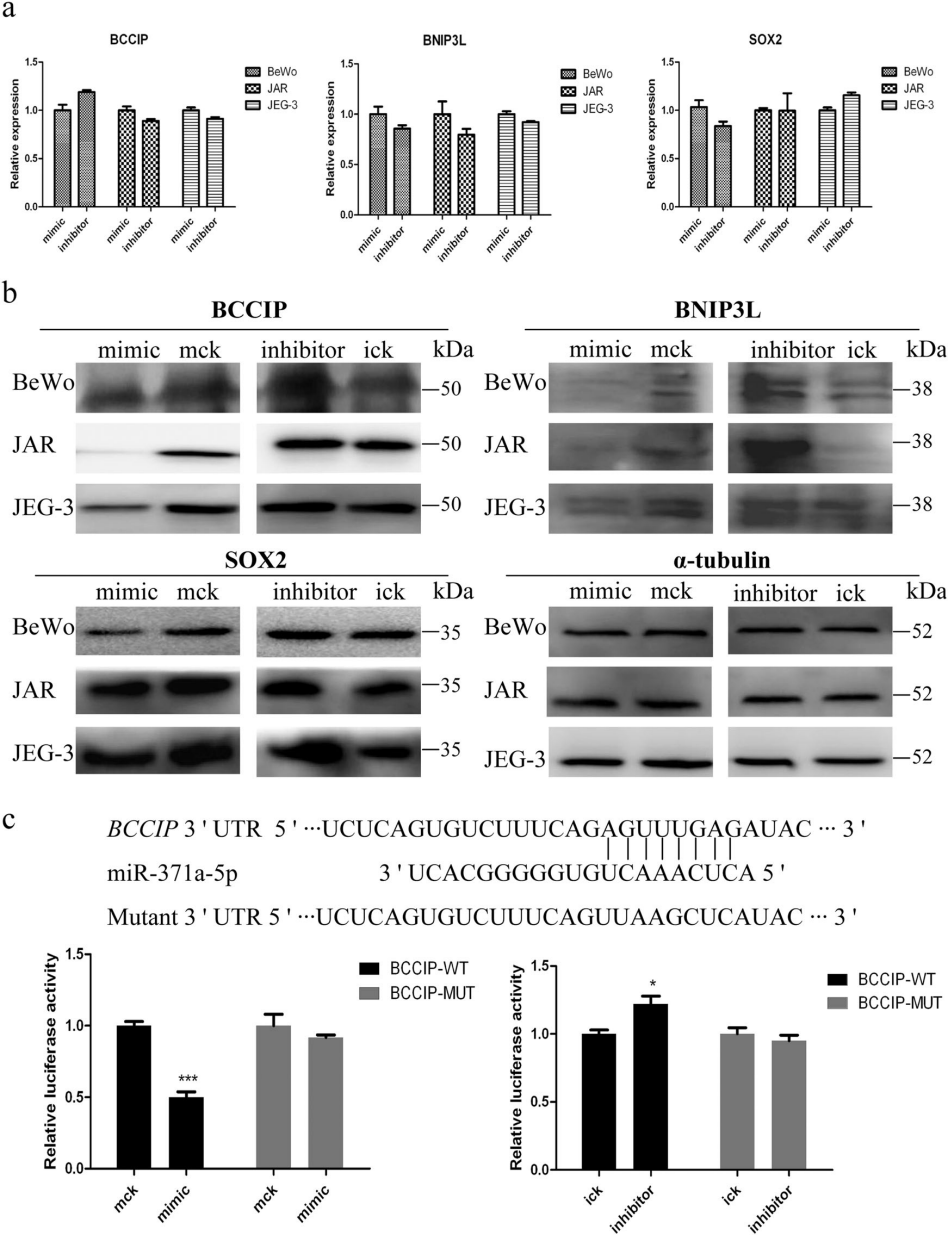
Validation of miR-371a-5p potential target genes *BCCIP*, *BNIP3L*, and *SOX2* **a** qRT-PCR quantification of the mRNA levels of *BCCIP*, *BNIP3L*, and *SOX2* genes. BeWo, JAR, and JEG-3 cells were transfected with miR-371a-5p mimic/inhibitor or control mimic/inhibitor. Cells were harvested at 36 h post transfection, and *α-tubulin* was used as the endogenous reference gene. **b** Western blotting analysis of BCCIP, BNIP3L, and SOX2. Cells were harvested at 48 h post transfection, and α-tubulin was used as the endogenous control. **c** Direct interaction of miR-371a-5p and *BCCIP*. Upper: bioinformatic predicted miR-371a-5p targeting site in 3′-UTR of *BCCIP*. Lower: luciferase activity assay. At 48 h post co-transfection with BCCIP–WT or BCCIP–MUT and miR-371a-5p mimic/inhibitor or control mimic/inhibitor, luciferase activities were measured in HTR-8/SVneo cells. *Renilla* luciferase activities in corresponding controls were normalized to 1. Mck stands for control mimic and ick stands for control inhibitor. *N* = 9, *P < 0.05, ***P < 0.001

**Fig. 6:**
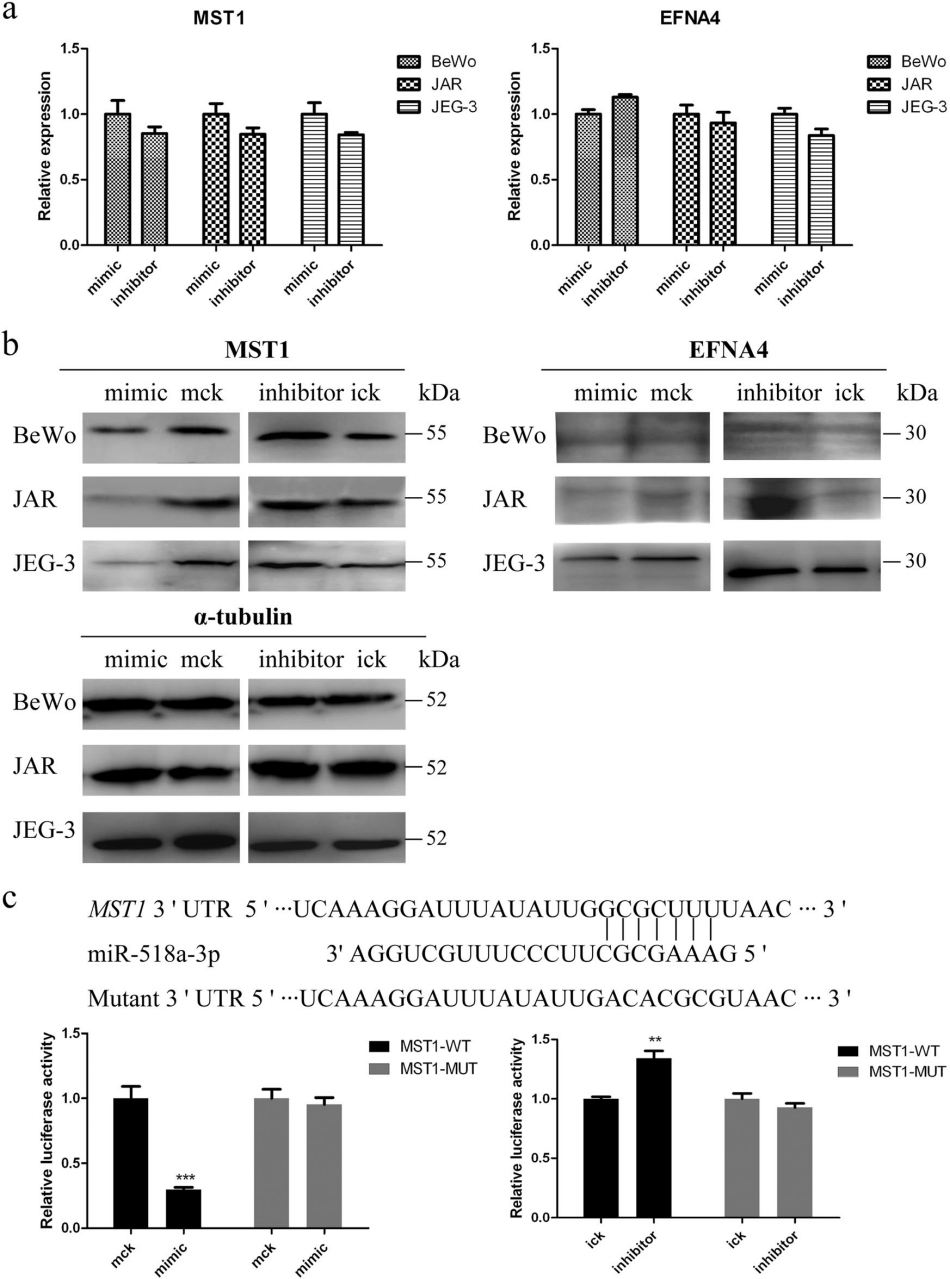
Validation of miR-518a-3p potential target genes *MST1* and *EFNA4* **a** qRT-PCR quantification of the mRNA level of *MST1* and *EFNA4* genes. BeWo, JAR, and JEG-3 cells were transfected with miR-518a-3p mimic/inhibitor or controls. Cells were harvested at 36 h post transfection, and *α-tubulin* served as the endogenous reference gene. **b** Western blotting analysis of MST1 and EFNA4. Cells were harvested at 48 h post transfection, and α-tubulin was taken as the endogenous control. **c** Direct interaction of miR-518a-3p and *MST1*. Upper: bioinformatic predicted miR-518a-3p targeting site in 3′-UTR of *MST1*. Lower: luciferase activity assay. At 48 h post co-transfection with MST1-WT or MST1-MUT and miR-518a-3p mimic/inhibitor or control mimic/inhibitor, luciferase activities were measured in HTR-8/SVneo cells. *Renilla* luciferase activities in corresponding controls were normalized to 1. Mck stands for control mimic and ick stands for control inhibitor. *N* = 9, **P < 0.01, ***P < 0.001

Given that miRNA function generally relies on their target genes, to further investigate regulation networks of miR-371a-5p and miR-518a-3p, their putative targets were predicted by TargetScan (http://www.targetscan.org), PicTar (http://pictar.mdc-berlin.de), miRanda (http://www.microrna.org), and miRecords (http://c1.accurascience.com/miRecords/). Based on enrichment analysis in Gene Ontology (GO) database, anti-oncogenes or genes encoding proteins with potential tumor-suppressing functions were figured out. *BCCIP*, *BNIP3L*, and *SOX2* were selected as representatives of miR-371a-5p downstream targets, while *MST1* and *EFNA4* were selected as representatives of miR-518a-3p downstream targets. BeWo, JAR, and JEG-3 cells were harvested at 36 h post miRNA mimics or inhibitors transfection. Then, qRT-PCR assay was carried out to investigate the alteration of transcripts of these genes. As indicated in Figs. 5a and 6a, overexpression of both miRNAs did not induce any significant change in these genes at mRNA level even compared with the knockdown groups. Meanwhile, western blotting demonstrated significantly decreased protein levels of BCCIP, SOX2, and MST1 in corresponding miRNAs overexpressed group, whereas exhibited a remarkable augmentation of these proteins in knockdown groups. However, the level of BNIP3L was rather low in all three cell lines, and only obvious elevated expression was observed in miR-371a-5p knockdown JAR cells. Besides, expression of EFNA4 was also low in BeWo and JAR cells, while significant alterations were only detected in miR-518a-3p knockdown JAR and JEG-3 cells (Figs. 5b and 6b). To verify the direct relationships of miR-371a-5p and miR-518a-3p with their potential target genes, wild-type and mutated 3′-UTR regions of *BCCIP* and *MST1* were constructed to a report vector as the representatives. To minimize the background, HTR-8/SVneo cells were used since the endogenous expressions of miR-371a-5p and miR-518a-3p were much lower than choriocarcinoma cells (Fig. 1b). Transient co-transfection with the wild-type 3′-UTR reporter plasmid BCCIP–WT and miR-371a-5p mimic led to a significant decrease in reporter activity (Fig. 5c). Meanwhile, significantly increased reporter activity was detected when BCCIP–WT co-transfected with miR-371a-5p inhibitor as compared with the control. Besides, reporter activities were almost unaffected when cells were co-transfected with the mutated 3′-UTR reporter plasmid BCCIP–MUT and miR-371a-5p mimic/inhibitor. Similar results were obtained when miR-518a-3p mimic/inhibitor co-transfected with MST1–WT or MST1–MUT (Fig. 6c).

### MiR-371a-5p and miR-518a-3p targeted multiple pathways in tumorigenesis and metastasis

**Fig. 7:**
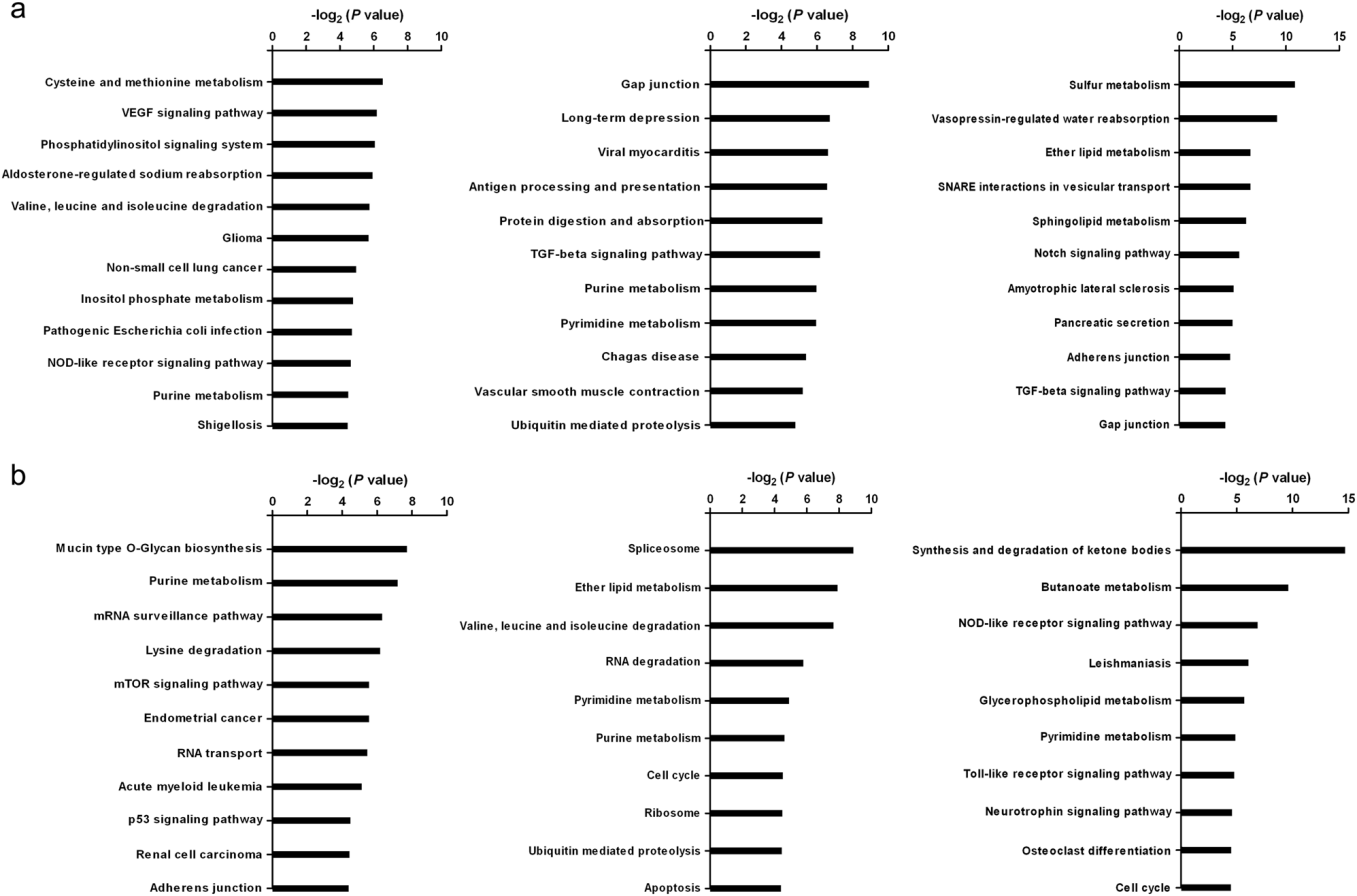
KEGG classification of the upregulated genes upon miR-371a-5p or miR-518a-3p knockdown BeWo, JAR, and JEG-3 cells were transfected with miR-371a-5p/miR-518a-3p inhibitors or control inhibitors. Transcriptomes were sequenced at 36 h post transfection, and the upregulated genes were identified with >2-fold changes and the adjusted *P* < 0.05. **a** Classification based on the upregulated genes after miR-371a-5p knockdown. Totally, 237, 132, and 277 genes were identified in BeWo, JAR, and JEG-3 cells, respectively. Left: BeWo cells; middle: JAR cells; right: JEG-3 cells. **b** Classification based on the upregulated genes after miR-518a-3p knockdown. Totally, 229, 269, and 191 genes were identified in BeWo, JAR, and JEG-3 cells, respectively. Left: BeWo cells; middle: JAR cells; right: JEG-3 cells

To identify the pathways and other differentially expressed genes (DEGs) that are potentially involved in miR-371a-5p and miR-518a-3p signaling, transcriptome sequencing was conducted in BeWo, JAR, and JEG-3 cells post miR-371a-5p and miR-518a-3p knockdown. A gene with a fragments per kilobase per million (FPKM) ratio larger than 2 or smaller than 0.5 and with the adjusted *P* value < 0.05 was considered to be a DEG. Totally, 453, 267, and 487 DEGs were identified in BeWo, JAR, and JEG-3 cells, respectively, after miR-371a-5p knockdown. Among which, 237, 132, and 277 genes were upregulated, respectively. After miR-518a-3p knockdown, 503, 605, and 563 DEGs along with 229, 269, and 191 upregulated ones were identified in BeWo, JAR, and JEG-3 cells, respectively. The top upregulated genes included many oncogenes or oncogenesis associated ones, such as *AKAP2*, *MYC*, and *IGF2* for miR-371a-5p, and *SNX22*, *CDKL5*, and *PTTG1IP* for miR-518a-3p (Table 1). Kyoto Encyclopedia of Genes and Genomes (KEGG) enrichment analysis of the upregulated genes also showed that miR-371a-5p and miR-518a-3p regulated diverse pathways related to tumorigenesis and metastasis, such as VEGF, TGF-β, and gap junction pathways for miR-371a-5p (Fig. 7a), and p53, cell cycle, and apoptosis pathways for miR-518a-3p (Fig. 7b).

**Table 1:** Top 10 upregulated genes induced by miR-371a-5p or miR-518a-3p knockdown in BeWo, JAR, and JEG-3 cells

Cells	Upregulated genes
miR-371a-5p knockdown	
BeWo	*AKAP2*, *CRAMP1*, *TMEM164*, *EBLN3*, *RAD54L2*, *YPEL2*, *MKL2*, *HNRNPA1L2*, *BLOC1S6*, *SPAST*
JAR	*CPSF6*, *EME2*, *TOMM6*, *MYC*, *GCH1*, *GFAP*, *UPK3BL*, *DAG1*, *ZBTB7B*, *ZKSCAN1*
JEG-3	*MATR3*, *SNX22*, *PEG10*, *PDP2*, *GOLGA8B*, *AKAP2*, *EBLN3*, *IGF2*, *TSN*, *IQGAP1*
miR-518a-3p knockdown	
BeWo	*IL6ST*, *SNX22*, *ZNF12*, *ZNF440*, *MPZL1*, *MOB1B*, *CHD2*, *ST6GAL1*, *YPEL2*, *SLC7A6*
JAR	*PEG10*, *ZDHHC20*, *SNX22*, *ZNF264*, *CDKL5*, *DCP2*, *SPAST*, *RASSF8*, *CLCN5*, *DYRK1A*
JEG-3	*SNX22*, *EME2*, *UPK3BL*, *EBLN3*, *TOMM6*, *RAB11FIP2*, *TMEM263*, *ZBTB43*, *PTTG1IP*, *KLHL23*

## Discussion

GTD is a range of pregnancy-related disorders. Some of the GTDs can further develop into life-threatening GTN. However, the molecular underpinnings driving GTD progression are largely unknown. MicroRNAs are regulators of genes generally at posttranscriptional level. Dysregulation of miRNAs is globally involved in tumorigenesis and cancer metastasis. Comparative analysis has revealed many differentially expressed miRNAs in cancers. In this study, we identified that miR-370-3p, -371a-5p, -518a-3p, -519d-3p, -520a-3p, and -934 were dysregulated in GTN. Although our microarray profiling data are still preliminary because of the small sample size, measurement of the six tissue sample-verified miRNAs would be helpful for the prognosis and clinical management of CHM patients. In this study, we took the >5-folds criterion for further verification in trophoblastic tissues. However, reports indicated that much smaller changes (<2-folds) could also be significant^23^. Therefore, we cannot exclude other possible hallmark miRNAs in CHM progression.

MiR-371a-5p is a member of the miR-371~373 cluster, which has been reported to be oncogenic, immune suppressive, and a stem cell signature^24^. However, studies about the functions and regulation networks of miR-371a-5p are very limited. Upregulation of miR-371a-5p was identified in the development of hepatic fibrosis^25^. MiR-371a-5p dysregulation was also reported to be involved in the gemcitabine-resistance in pancreatic cancer cells^26^ and radioresistance in nasopharyngeal carcinoma cells^27^. MiR-518a-3p is a member of the primate-specific C19MC, which accounts for about 8% of all known human miRNA genes. C19MC plays important roles in cell differentiation and immunomodulation during pregnancy^28^. Overexpression of miR-518a-3p has been detected in hepatocellular carcinoma, breast cancer, and Sezary syndrome. The metastatic foci in the lymph nodes of some tumors also showed upregulated miR-518a-3p compared with primary tumors^29^. Additionally, Port et al. reported that miR-518a-3p was upregulated in cisplatin-resistant germ cell tumor cell lines^30^. Nonetheless, reports about miR-518a-3p are diverse among different cancers. A study indicated that miR-518a-3p was downregulated in colorectal cancer cell lines and tissues with metastases^31^. Xu et al. also reported that miR-518a-3p was downregulated in chemo-resistant breast cancer cells^32^. Our study demonstrated that overexpressions of miR-371a-5p and miR-518a-3p promoted proliferation, migration, and invasion of choriocarcinoma cells. Meanwhile, knockdown of both miRNAs upregulated many genes in diverse pathways related to tumorigenesis and metastasis. Besides, many downregulated genes post miR-371a-5p and miR-518a-3p knockdown were associated with interferon responses. GO analysis of all the DEGs revealed that miR-371a-5p was mainly associated with phosphatidylinositol binding, TGF-β response, interferon response, and apoptosis (Supplementary Figure S6a) while miR-518a-3p was mainly associated with immune response (Supplementary Figure S6b). All these findings reinforced the pivotal “oncomiRNAs” and immunomodulator roles of miR-371a-5p and miR-518a-3p in the progression of GTD. Moreover, crosstalk between these two miRNAs cannot be excluded since a particular miRNA can modulate multiple pathways.

Generally, the regulatory function of miRNA depends on its target genes. However, studies about the target genes of miR-371a-5p and miR-518a-3p are few. One study reported that BAG3, a component of the chaperone-assisted autophagy pathway, was a direct target of miR-371a-5p^33^. Another study proved that NIK, a regulator of NF-κB, was a direct target of miR-518a-3p^31^. In the current study, we demonstrated that miR-371a-5p negatively regulated BCCIP, BNIP3L, and SOX2, and miR-518a-3p negatively regulated MST1 and EFNA4. Importantly, we proved that *BCCIP* was a direct target of miR-371a-5p, and *MST1* was a direct target of miR-518a-3p for the first time. Therefore, miR-371a-5p and miR-518a-3p may drive the pathogenesis of GTN via regulating these tumor suppressors. However, in our transcriptome sequencing, only 4 genes, including *PEG10*, *CYB561D1*, *TCF12*, and *SUOX*, were commonly upregulated in BeWo, JAR, and JEG-3 cells after miR-371a-5p knockdown. Meanwhile, *SNX22*, *HMGCS1*, and *NBPF12* were commonly upregulated after miR-518a-3p knockdown. Two factors may contribute to such a few commonly upregulated genes. One is possibly the genetic difference of those three cell lines. Our principal component analysis of the transcriptomes revealed that BeWo cells shared only about 80% and 83% similarities with JAR and JEG-3 cells, respectively, while JAR cells shared about 83% similarity with JEG-3 cells. Another factor is possibly the intrinsic regulation mechanisms of miRNA. Numerous reports indicated that miRNA could repress many of their targets without detectable changes in mRNA levels^34^. Thus, the >2-folds criteria in our data processing might be too stringent. The exact roles of those commonly identified genes in the progression of GTD still need to be clarified.

BCCIP, a BRCA2 and p21^Waf1/Cip1^ cofactor, is abundantly expressed in many tissues. It reaches its highest level at the S/G1 border and remains high in the S phase^35^. BCCIP is also required for the transactivation activity of p53^36^. BNIP3L, a mitochondrial protein, has been revealed to be a proapoptotic transcriptional target of p53^37^. Overexpresison of BCCIP can inhibit S to G1 progression, whereas downexpresison of BCCIP leads to chromosomal polyploidization and centrosome amplification^38^. Therefore, BCCIP may be involved in the miR-371a-5p-mediated proliferation of choriocarcinoma cells. SOX2 is a core transcription factor in the maintenance of the undifferentiated phenotype of stem cells. Our previous studies also have reported that SOX2 was downregulated in HM, choriocarcinoma tissues, and choriocarcinoma cells^39^. Naturally, cytotrophoblasts own certain stem cell characteristics. Thus, downregulation of SOX2 in GTN might induce the alteration of the controlled normal self-renewal of cytotrophoblasts to facilitate the tumorigenesis.

MST1 is a highly conserved Ser/Thr kinase, acting as a tumor suppressor by restricting cell proliferation and survival. Loss or inactivation of MST1 has been found in many tumors, including colorectal cancer, hepatocellular carcinoma, and soft-tissue sarcoma^40,41^. Apart from the functions in apoptosis signaling, studies indicated that MST1 was also implicated in centrosome duplication and mitotic chromosome alignment^42^, which may explain our observation of the S-phase arrest by miR-518a-3p inhibitor. The effect of MST1 is reported to be opposite to that of AKT^43^, consistent with our previous report that the activated AKT signaling pathway was essential for the pathogenesis of GTD^44^. We thus assume that there may be a miR-518a-3p–MST1–AKT regulatory axis in the progression of GTN. Eph-ephrin system has been demonstrated to play important roles in migration of trophoblasts and in the initial step of embryo implantation. Nonetheless, studies about the function of EFNA4 in trophoblasts are few; only one report indicated that recombinant human ephrin A4 could promote the invasion of JEG-3 cells^45^. In this study, the low expression of EFNA4 suggests that it may not play a major role in miR-518a-3p-mediated metastasis of choriocarcinoma cells. Collectively, these findings suggest that miR-371a-5p and miR-518a-3p regulate the proliferation, migration, and invasion processes by interacting with diverse targets in trophoblasts, thereby affecting the pathogenesis of GTN.

In summary, we identified that miR-370-3p, -371a-5p, -518a-3p, -519d-3p, -520a-3p, and -934 were differentially expressed in GTN vs. CHM. We demonstrated that miR-371a-5p and miR-518a-3p led to the aggressive characteristics of trophoblastic cells by regulating BCCIP, SOX2, and MST1. Our findings provide useful insight into the investigation of diagnostic biomarkers and therapeutic targets for GTN.

## Materials and methods

### Tissue samples and ethics statement

A total of 62 FFPE trophoblastic blocks, including 6 normal first-trimester placentas, 35 regressed CHM cases (CHMs), and 21 progressed CHM cases (GTN) after uterine evacuation, were retrieved from the bio-bank of International Peace Maternity and Child Health Hospital (IPMCH), Shanghai Jiao Tong University. Fresh trophoblastic tissues, including 6 cases of normal first-trimester placentas and 38 cases of CHMs (25 regressed ones), were also collected from the bio-bank of IPMCH. Every sample was diagnosed according to the morphological and clinical criteria^1,2^. Informed consent was obtained from each patient recruited. Ethical approval for the use of these tissues in this study was obtained from the IPMCH ethical review board.

### Cell culture

Human choriocarcinoma cell lines BeWo, JAR, and JEG-3 were obtained from Shanghai Cell Bank of Chinese Academy of Sciences (China), and were characterized by DNA fingerprinting and isoenzyme analysis. These cells were maintained in Dulbecco’s modified Eagle’s medium (DMEM, Invitrogen, NY, USA) supplemented with 10% fetal bovine serum (FBS, Invitrogen) at 37 °C. HTR-8/SVneo, an immortalized normal trophoblastic cell line, was cultured in RPMI 1640 medium (Invitrogen) supplemented with 10% FBS. Villous primary human trophoblasts (PHT) were isolated from first-trimester placentas as described previously^46^ and were cultured in DMEM/F12 medium (Invitrogen) supplemented with 15% FBS.

### miRNA array

Total RNA was extracted from fresh trophoblastic tissues with Trizol reagent (Invitrogen). RNA concentration was determined by Nanodrop 2000c (Thermo Fisher Scientific, Waltham, MA, USA). RNA integrity was evaluated by using an Agilent 2100 Bioanalyzer (Agilent, Santa Clara, CA, USA). MiRNA was then purified with mirVana™ miRNA Isolation Kit (Thermo Fisher Scientific). After Poly(A) tailing and biotin labeling, hybridization was conducted on Affymetrix GeneChip miRNA 4.0. Data were obtained and analyzed with AGCC software (Affymetrix, Santa Clara, CA, USA).

### Quantitative RT-PCR

Cells were washed three times with corresponding serum-free culture medium before harvesting. Total RNA was extracted from fresh trophoblastic tissues or cells with Trizol reagent. Extraction of miRNA from FFPE tissues was carried out with miRNeasy FFPE Kit (Qiagen, Hilden, Germany). For mRNA analysis, cDNA synthesis was carried out by using the PrimeScript 1st Strand cDNA Synthesis Kit (Takara, Dalian, China) and qRT-PCR was carried out by using the SYBR qRT-PCR Kit (Takara). For miRNA quantification, miRNA cDNA 1st Strand Kit and miRNA qPCR Assay Kit (CWBIO, Beijing, China) were used along with gene-specific primers. All qRT-PCR reactions were performed in triplicate on an ABI StepOneplus system (Applied Biosystems, Foster, CA, USA). *α-tubulin* was used as the endogenous reference gene for mRNA analysis. *RNU6* was used as the reference gene for miRNA analysis. Relative mRNA or miRNA level was calculated by using the 2^-ΔΔCt^ method.

### ISH and fluorescence in situ hybridization (FISH)

ISH and FISH were carried out on 6-µm-thick FFPE sections. Two double-digoxigenin (DIG)-labeled oligonucleotide probes, complementary to mature miR-371a-5p (Exon Biotechnology, Guangzhou, China) and miR-518a-3p (Genscript, Nanjing, China), respectively, were synthesized. An *RNU6*-specific probe was used as the positive control. After deparaffinization, hydration, and proteinase K digestion, 40 pmol probe was applied for each section. Hybridization was carried out at 55 °C for 1 h in a Leica hybridizer (Wetzlar, Germany), and then washed in a decreasing gradient of SSC buffers at 55 °C. Next, sections were incubated for 15 min at 37 °C with blocking solution, and then with sheep anti-DIG-AP antibody (1:200 diluted, Boster, Wuhan, China) for 1 h at 37 °C. Finally, sections were visualized with chromogene NBT/BCIP (Boster) and nuclei were stained with nuclear fast red (Boster). All sections were scored by three pathologists independently and blindly, and dominant staining intensity was scored as follows: negative (−), weak (+), intermediate (++), and strong (+++). For FISH, the sheep anti-DIG-AP antibody was replaced with sheep anti-DIG-FITC antibody (1:100 diluted, Boster) and nuclei were stained with DAPI (Boster). Sections were analyzed under a Leica microscope with LAS v4.2 software.

### Transfection of the miRNA mimic and inhibitor

MiRNA mimic/inhibitor and the control mimic/inhibitor were synthesized by RiboBio Company (Guangzhou, China). MiRNA mimics are double-stranded RNA molecules that mimic endogenous mature miRNA whereas miRNA inhibitors are single-stranded nucleic acids that specifically bind and inhibit endogenous miRNA^47^. Choriocarcinoma cells were plated in 24-well plates the day before transfection and grown to 70–80% confluence. Cells were then transfected with 150 nM inhibitor or 100 nM mimic by using Lipofectamine 3000 (Invitrogen) according to the manufacturer’s instructions.

### Cell proliferation assay

BeWo, JAR, and JEG-3 cells were seeded in 96-well plates at 1 × 10^4^ cells per well density the day before transfection. Twenty-four hours post transfection with 30 nM mimic or 40 nM inhibitor, cell viability was measured every 12 or 24 h with Cell Counting Kit 8 reagent (DOJINDO, Kumamoto, Japan). Optical density was read at 450 nm using a universal microplate reader (Bio-Tek, Winooski, VT, USA).

### Cell cycle analysis

About 1 × 10^6^ cells were collected at 48 h post transfection. After washing twice with ice-cold PBS, cells were fixed in precooled 70% ethanol at −20 °C overnight. Before analysis by flow cytometry, cells were washed three times with PBS, resuspended in 500 µl propidium iodide/RNase staining solution (BD Biosciences, San Jose, CA, USA), and incubated in the dark for 30 min at 37 °C. The percentage of cells in each phase of cell cycle was calculated by using the Multicycle Program (Beckman Coulter, Brea, CA, USA).

### Cell migration and invasion assays

Cell migration was detected by using transwell assay. Briefly, at 24 h post transfection, cells were pretreated with mitomycin C (Selleck, Shanghai, China) for 2 h. Then, 5 × 10^4^ cells suspended in 100 μl serum-free DMEM were seeded into the transwell insert with 8-µm-diameter pore membrane (Corning, Shanghai, China). As a chemoattractant, 500 μl DMEM medium containing 15% FBS was added into the lower chamber. After incubation at 37 °C for 36 h, non-migrated cells on the upper surface of the membrane were removed with cotton swabs. Cells that attached to the undersurface of the membrane were fixed with 4% paraformaldehyde for 30 min, and then stained with 0.1% crystal violet for 15 min. For invasion assay, the upper chamber was coated with 100 µl Matrigel (BD Biosciences) before incubation. Then, 1 × 10^5^ cells were seeded and incubated for 48 h at 37 °C. Invaded cells were fixed and stained following the same protocol as the migration assay. Cells were counted under a light microscope (Olympus, Tokyo, Japan) at 100× magnification, and five randomly selected fields were scored per well. Mean cell number of the five fields was calculated to be the number of migrated or invaded cells for that well. Both assays were carried out in triplicate.

### Western blotting

Cells were harvested at 48 h post transfection, and lysed in SDS lysis buffer (Beyotime, Beijing, China) with fresh addition of 1% protease inhibitor cocktail (Sigma-Aldrich, Darmstadt, Germany) and 1 mM PMSF (Sigma-Aldrich). Protein concentrations were determined by the Bradford method with the BCA Protein Assay kit (Thermo Fisher Scientific). Equal amount of proteins (30 µg) were resolved in 10% SDS-PAGE and then transferred to PVDF membranes (Millipore, Billerica, MA, USA). After blocking, PVDF membranes were washed and incubated with different primary antibodies [1:1000 diluted mouse anti-BCCIP (08269, Sigma-Aldrich), rabbit anti-BNIP3L (12,396, Cell signaling technology, Danvers, MA, USA), rabbit anti-SOX2 (3579, Cell signaling technology), goat anti-MST1 (AF4949, R&D systems, Minneapolis, MN, USA), goat anti-EFNA4 (AF369, R&D systems) antibodies, 1:5000 diluted rabbit anti-α-tubulin monoclonal antibody (2125, Cell signaling technology)] at 4 °C overnight. After re-warming for 30 min at room temperature and three times washing, membranes were incubated with HRP-linked goat anti-rabbit, goat anti-mouse, or rabbit anti-goat IgG (1:5000 diluted, Jackson ImmunoResearch, West Grove, PA, USA) for 1 h at 37 °C. Protein bands were developed by using the ECL kit (Tiangen Biotech, Beijing, China), and images were captured with Amersham Imager 600 (GE Healthcare, Chicago, IL, USA).

### Luciferase reporter assays

Wild-type or mutated 3′-UTR regions of *BCCIP* and *MST1* were cloned into the psiCHECK-2 vector (Promega, Madison, WI, USA) and nominated as BCCIP–WT, BCCIP–MUT, MST1–WT, and MST1–MUT, respectively. Then, HTR-8/SVneo cells were seeded in 24-well plates the day before transfection and grown to 70% confluence. Plasmids (400 ng) were co-transfected with corresponding miRNA mimic/inhibitor or control mimic/inhibitor (100 nM) by using Lipofectamine 3000 reagent. At 48 h post transfection, cells were lysed and luciferase activity was measured by using the Dual-Luciferase Reporter Assay System (Promega). *Renilla* luciferase signal was normalized to firefly luciferase signal. Each assay was performed in triplicates and three parallel wells were set for each time.

### Transcriptome sequencing

BeWo, JAR, and JEG-3 cells were transfected with miR-371a-5p or miR-518a-3p inhibitors or control inhibitor. At 36 h post transfection, cells were washed three times with PBS. Total RNA was extracted with the RNeasy Mini Kit (Qiagen). RNA concentration was determined by Nanodrop 2000c. RNA integrity was evaluated by using an Agilent 2100 Bioanalyzer. Poly(A) RNA were then purified with oligo(dT) beads and used for cDNA library generation and paired-end transcriptome sequencing (ShanghaiBio, Shanghai, China). Sequencing was performed using an Illumina HiSeq 2500 (San Diego, CA, USA) with more than 6 G bases throughput for every sample. Reads were aligned to the human genome (http://www.ensembl.org/Homo_sapiens/Info/Index) using HISAT2^48^. Data were normalized as FPKM. DEGs were identified with an adjusted *P* value <0.05 and a fold change >2. GO (http://www.geneontology.org/) and KEEG (http://www.genome.jp/kegg/) pathway enrichment analyses were then performed based on DEGs or upregulated genes.

### Statistical analysis

All experiments were performed at least in triplicates. Data were presented by mean ± SD where applicable. Differences were evaluated by using Mann–Whitney *U*-test, χ^2^ test, and *t*-test for two-group comparisons, while one-way ANOVA, Kruskal-Wallis, and χ^2^ tests for multiple comparisons. Statistical analyses were performed using R 3.1.3 (Lucent Technologies, Murray Hill, NJ, USA), SPSS 14.0 (IBM, Armonk, NY, USA), or GraphPad Prism 5 (GraphPad Software, La Jolla, CA, USA) software packages. The probability of *P* < 0.05 was considered to be statistically significant.

## Electronic supplementary material

supplemental Table 1

supplemental Table 2

supplemental Figure 1

supplemental Figure 2

supplemental Figure 3

supplemental Figure 4

supplemental Figure 5

supplemental Figure 6

Supplementary Figures
