# Supplementary material for: Identification of microRNA signature in the progression of gestational trophoblastic disease
Source: Cell Death Dis. 2018 Jan 24;9(2):94. doi: 10.1038/s41419-017-0108-2 (PMC5833456; doi:10.1038/s41419-017-0108-2)
Supplement: Supplementary file 1 — supplemental Table 1 [file 41419_2017_108_MOESM1_ESM.doc]

**Table S1. The expression of miR-371a-5p in** **FFPE tissues of CHM and GTN by *in situ* hybridization**.

| Group | Total cases | Grade | | | |
| --- | --- | --- | --- | --- | --- |
|  |  | - | + | ++ | +++ |
| CHM | 35 | 3 | 20 | 10 | 2 |
| GTN | 21 | 0 | 4 | 3 | 14 |
