# Supplementary figures and images for: Identification of microRNA signature in the progression of gestational trophoblastic disease
Source: Cell Death Dis. 2018 Jan 24;9(2):94. doi: 10.1038/s41419-017-0108-2 (PMC5833456; doi:10.1038/s41419-017-0108-2)

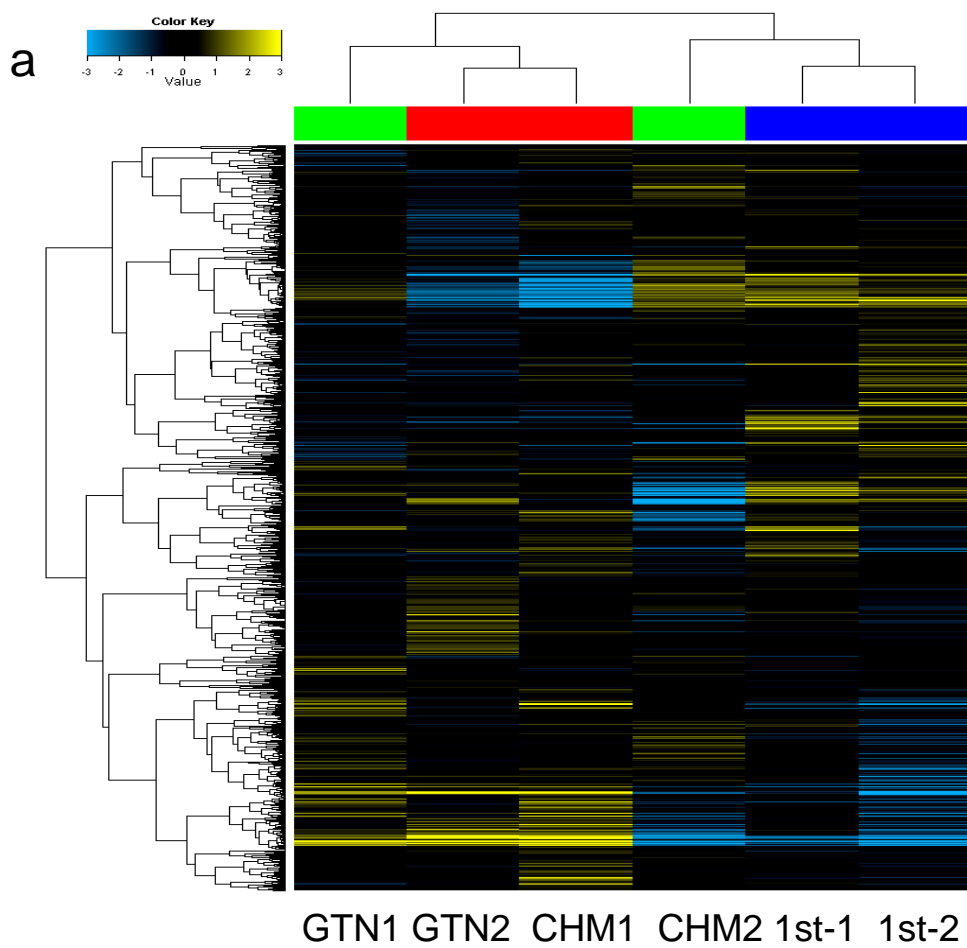

**b**

Log-Log Scatter Plot

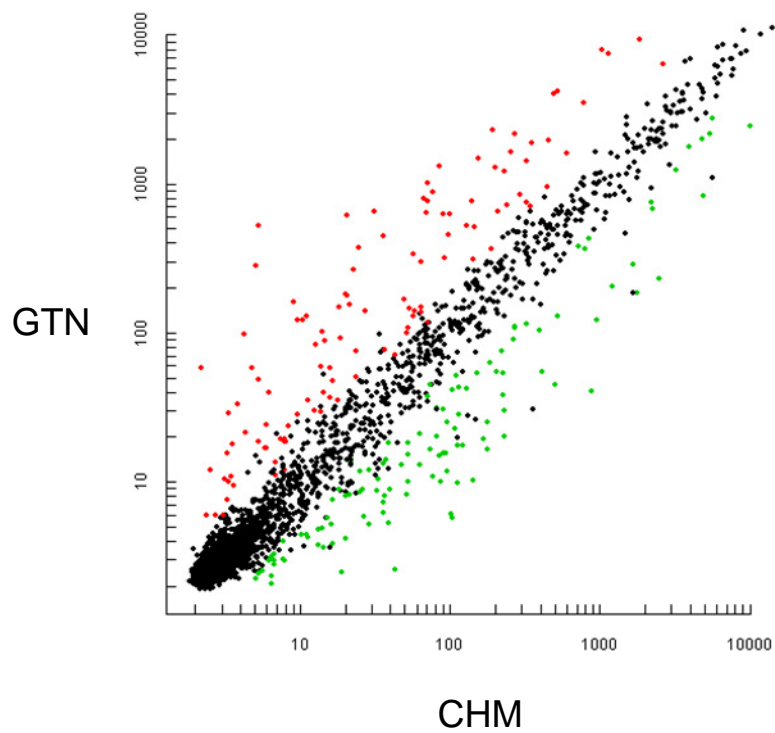

Supplement: Supplementary file 3 — supplemental Figure 1 [file 41419_2017_108_MOESM3_ESM.pdf]

miR-371a-5p

miR-518a-3p

normal first  
trimester  
placentas

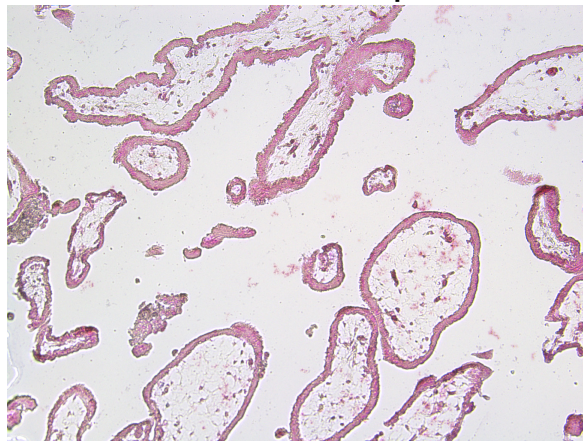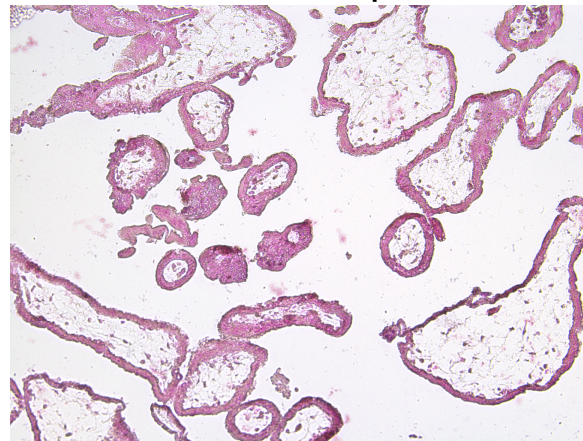

CHM

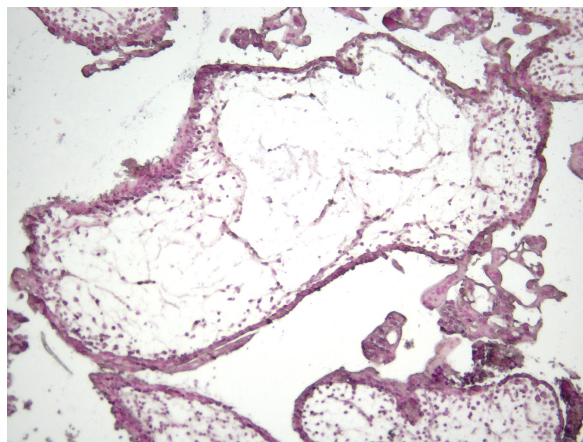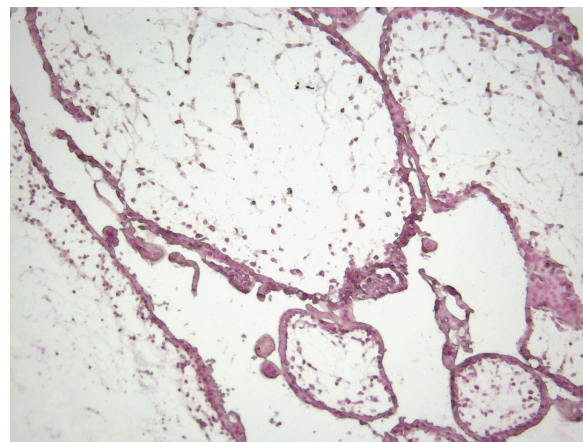

GTN

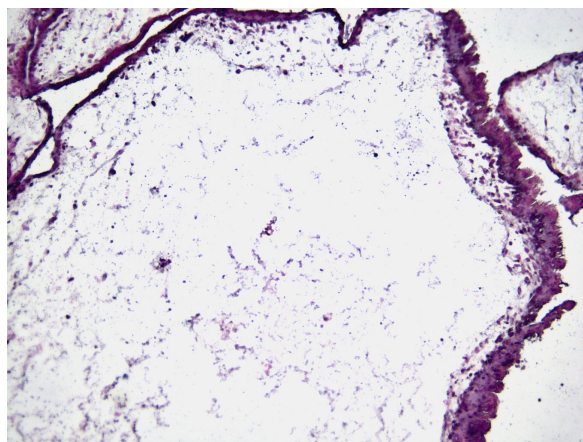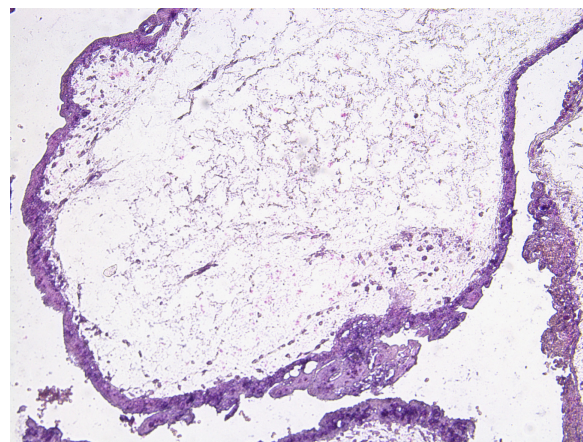

Supplement: Supplementary file 4 — supplemental Figure 2 [file 41419_2017_108_MOESM4_ESM.pdf]

a

BeWo

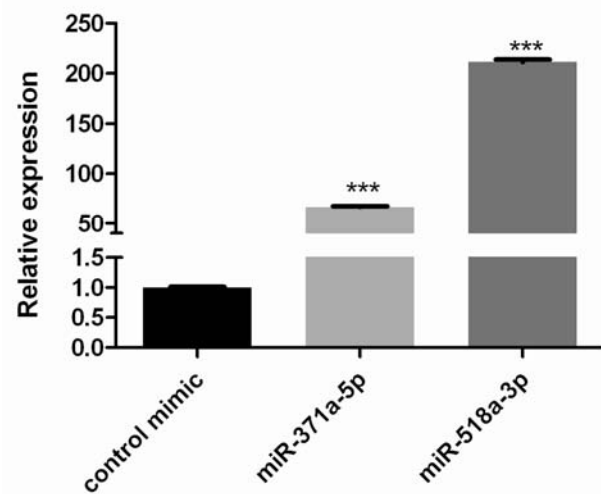

JAR

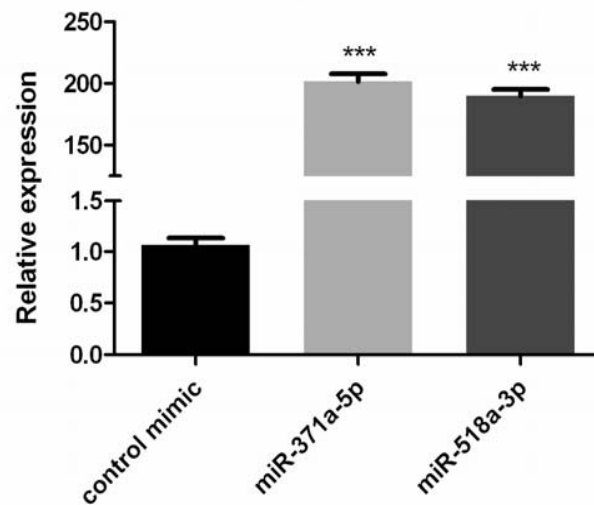

JEG-3

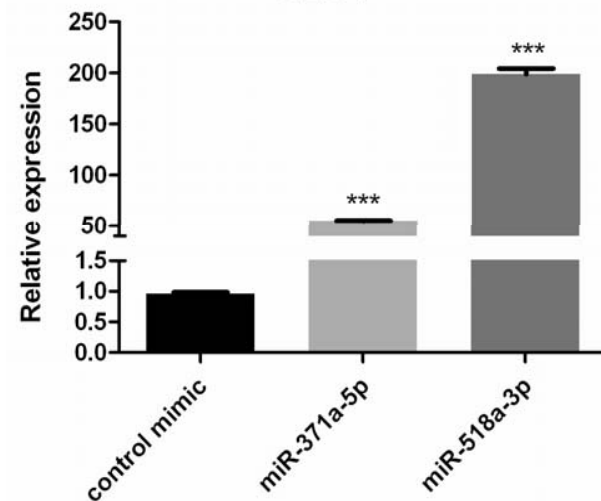

b

BeWo

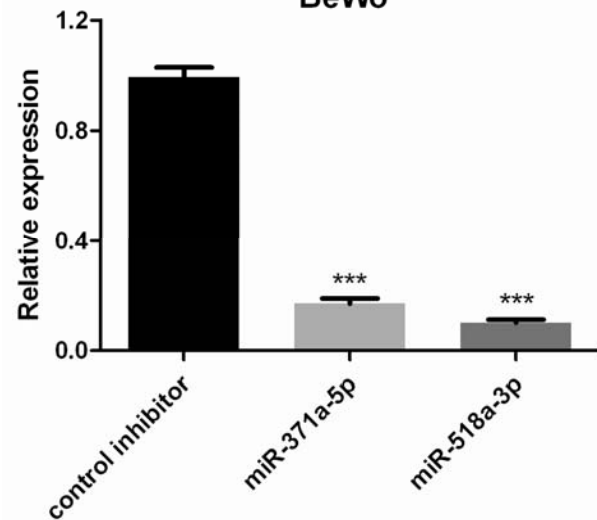

JAR

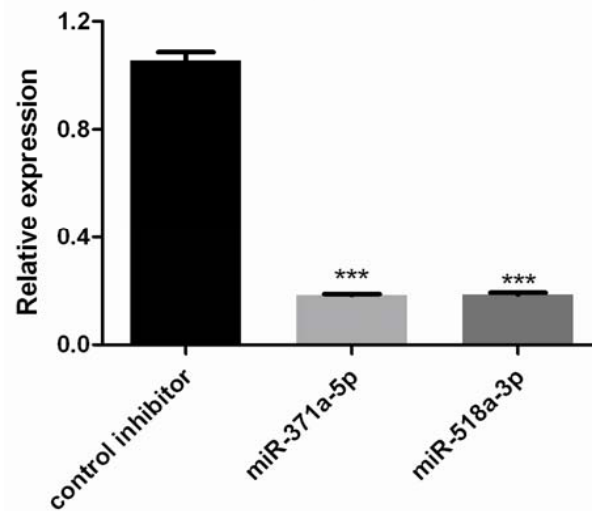

JEG-3

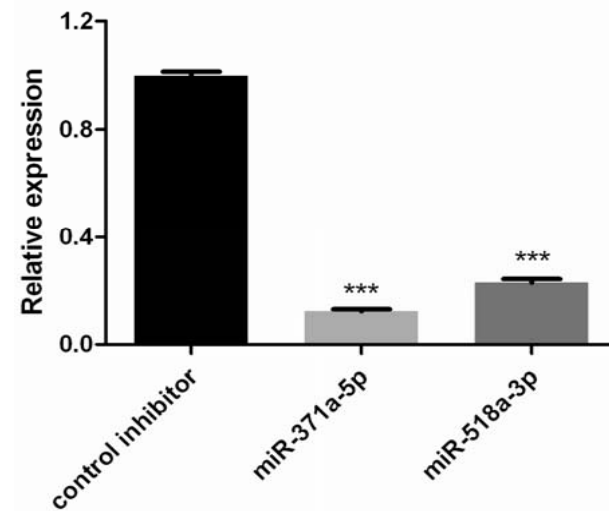

Supplement: Supplementary file 5 — supplemental Figure 3 [file 41419_2017_108_MOESM5_ESM.pdf]

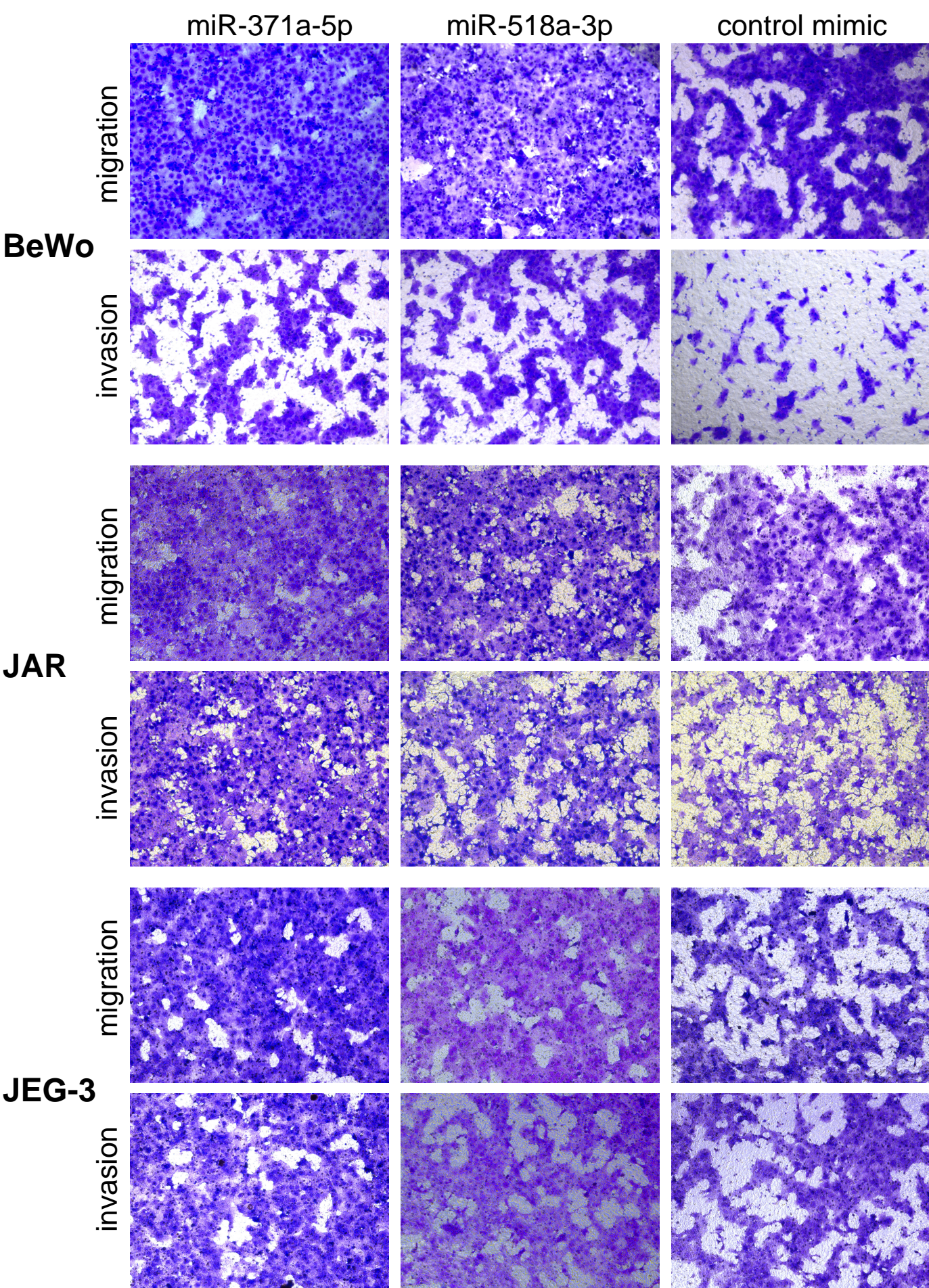

Supplement: Supplementary file 6 — supplemental Figure 4 [file 41419_2017_108_MOESM6_ESM.pdf]

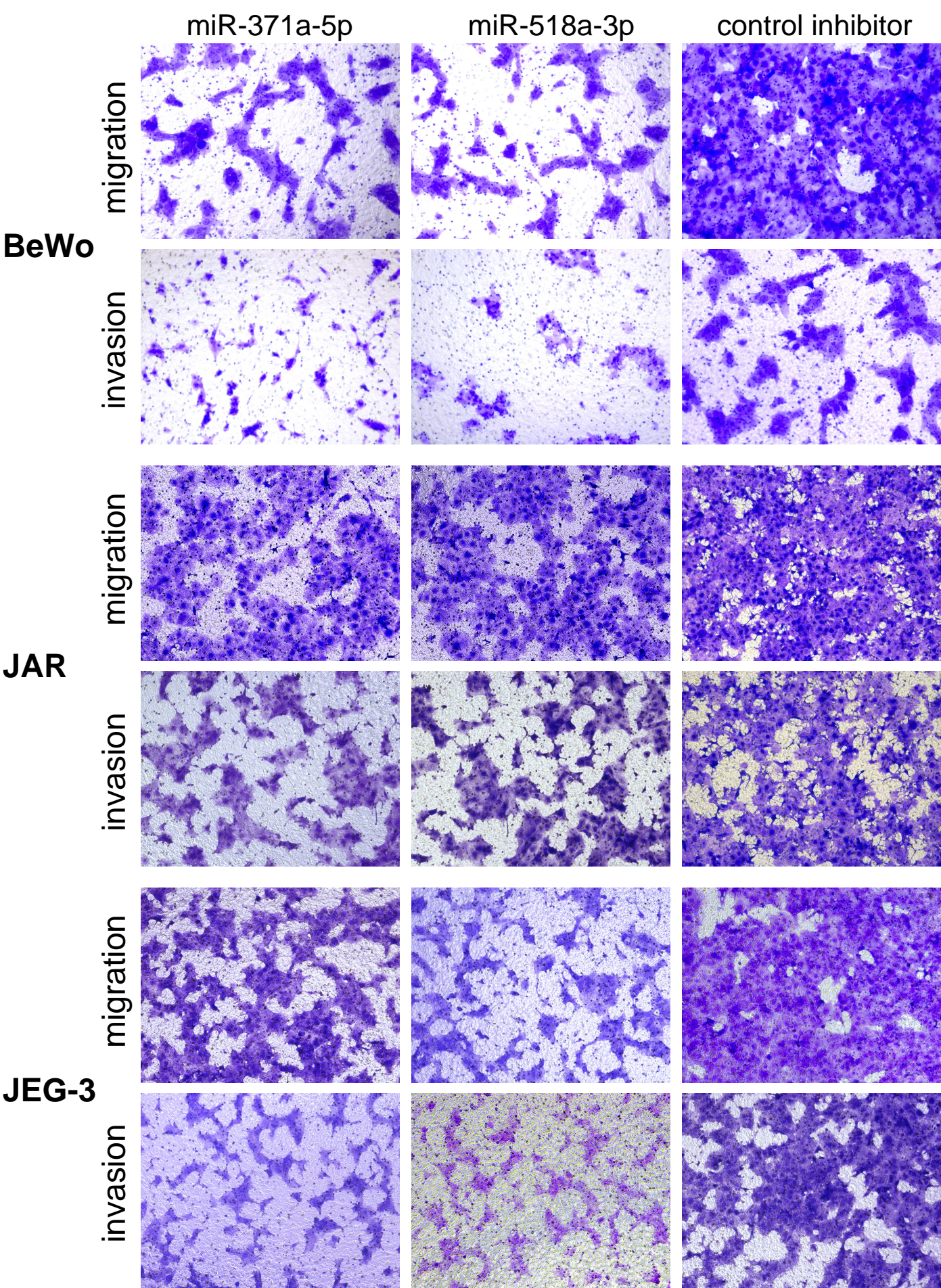

Supplement: Supplementary file 7 — supplemental Figure 5 [file 41419_2017_108_MOESM7_ESM.pdf]

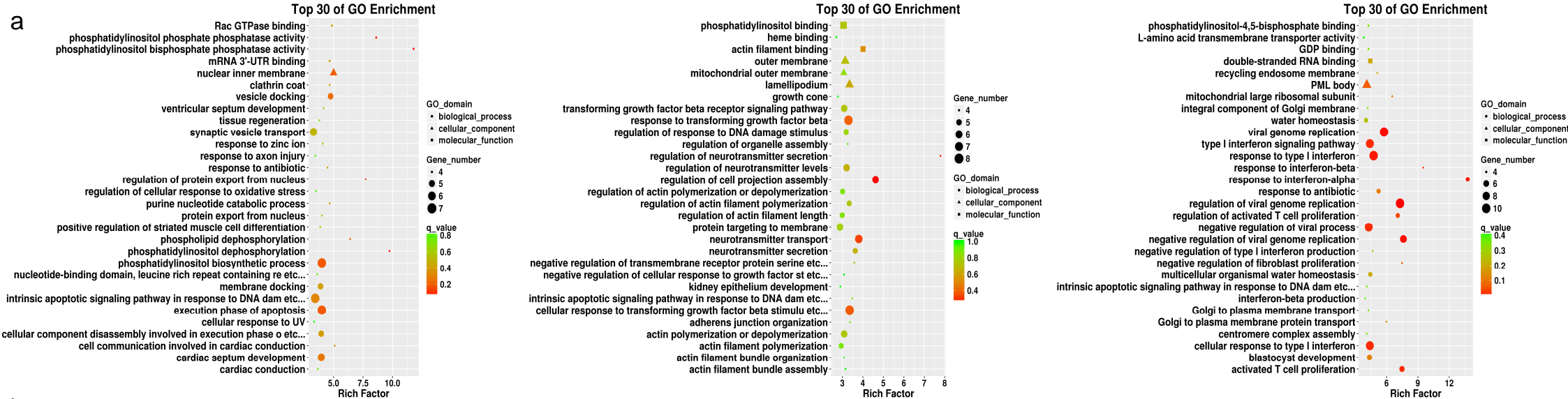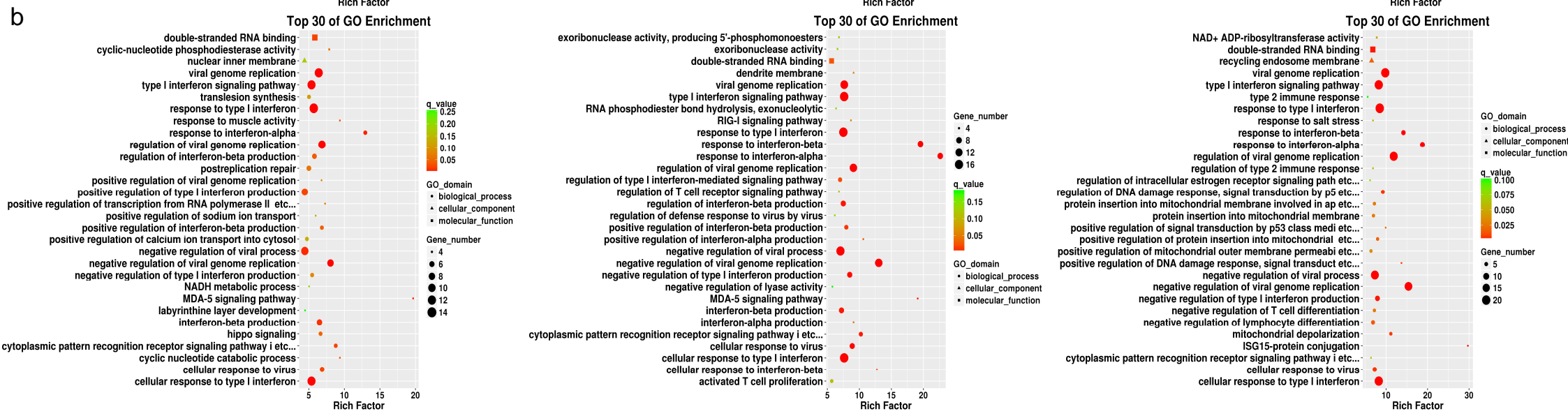

Supplement: Supplementary file 8 — supplemental Figure 6 [file 41419_2017_108_MOESM8_ESM.pdf]
