# Supplementary material for: Identification of microRNA signature in the progression of gestational trophoblastic disease
Source: Cell Death Dis. 2018 Jan 24;9(2):94. doi: 10.1038/s41419-017-0108-2 (PMC5833456; doi:10.1038/s41419-017-0108-2)
Supplement: Supplementary file 9 — Supplementary Figures [file 41419_2017_108_MOESM9_ESM.docx]

**Supplementary figure legends**

**Figure S1. MiRNA expression profiles discriminated GTN, CHM and normal first trimester placental tissues.**

A miRNA microarray was used to evaluate the miRNA expression profiles in tissues of GTN, CHM and normal first trimester placentas (1st) (two cases each). (a) Hierarchical clustering of differentially expressed miRNAs. Clustering tree was generated with Cluster 3.0 software. Blue color indicates lower expression; yellow color indicates higher expression. (b) Scatter plot of miRNAs profiles in tissues of GTN and CHM. Diagram was generated with R 3.1.3 software.

**Figure S2. Representative micrographs of miR-371a-5p and miR-518a-3p detected with *in situ* hybridization (ISH).**

ISH was performed on FFPE trophoblastic blocks, including 6 normal first trimester placentas, 35 CHM cases and 21 GTN cases. Positive staining signal was developed with NBT/BCIP (violet). Nuclei were counterstained with nuclear fast red. Magnification 100 ×.

**Figure S3. Evaluation of transfection efficiencies.**

BeWo, JAR and JEG-3 cells in 24-well plates were transfected with 100 nM miR-371a-5p or miR-518a-3p mimics (a) or 150 nM inhibitors (b). Expressions of corresponding miRNAs were quantified by qRT-PCR and normalized to *RNU6* at 24 h post-transfection. Relative expressions were normalized against corresponding controls. N = 3, *** *P* < 0.001.

**Figure S4. MiR-371a-5p and miR-518a-3p overexpressions promoted migration and invasion of choriocarcinoma cells.**

BeWo, JAR and JEG-3 cells were transfected with miR-371a-5p or miR-518a-3p mimics. Cells were pretreated with mitomycin C and then harvested and seeded into transwell inserts with (for invasion) or without (for migration) Matrigel at 24 h post transfection. After incubation, cells were fixed and stained, and then visualized at 100 × magnification.

**Figure S5. Silencing of miR-371a-5p and miR-518a-3p inhibited migration and invasion of choriocarcinoma cells.**

BeWo, JAR and JEG-3 cells were transfected with miR-371a-5p or miR-518a-3p inhibitors. Cells were pretreated with mitomycin C and then harvested and seeded into transwell inserts with (for invasion) or without (for migration) Matrigel at 24 h post transfection. After incubation, cells were fixed and stained, and then visualized under at 100 × magnification.

**Figure S6. GO classification of the differentially expressed genes (DEGs) upon miR-371a-5p or miR-518a-3p inhibition.**

BeWo, JAR and JEG-3 cells were transfected with miR-371a-5p/miR-518a-3p inhibitors or control inhibitor. Transcriptomes were sequenced at 36 h post transfection, and the DEGs were identified with the > 2 folds changes and the adjusted *P* < 0.05. (a) Classification based on the DEGs after miR-371a-5p knockdown. Totally, 453, 267 and 487 DEGs were identified in BeWo, JAR and JEG-3 cells respectively. Left: BeWo cells; middle: JAR cells; right: JEG-3 cells. (b) Classification based on the DEGs after miR-518a-3p knockdown. Totally, 503, 605 and 563 DEGs were identified in BeWo, JAR and JEG-3 cells respectively. Left: BeWo cells; middle: JAR cells; right: JEG-3 cells. *Q* value stands for adjusted *P* value.
